# The Endless Challenges of Arboviral Diseases in Brazil

**DOI:** 10.3390/tropicalmed5020075

**Published:** 2020-05-09

**Authors:** Tereza Magalhaes, Karlos Diogo M. Chalegre, Cynthia Braga, Brian D. Foy

**Affiliations:** 1 Arthropod-Borne and Infectious Diseases Laboratory (AIDL), Department of Microbiology, Immunology and Pathology (MIP), Colorado State University, Fort Collins, CO 80523-1692, USA; Brian.Foy@colostate.edu; 2 Oswaldo Cruz Institute (IOC), Oswaldo Cruz Foundation (FIOCRUZ), Vice Presidency of Production and Innovation in Health (VPPIS), Rio de Janeiro, RJ 21040-900, Brazil; diogochalegre@gmail.com; 3 Aggeu Magalhaes Institute (IAM), Oswaldo Cruz Foundation (FIOCRUZ), Department of Parasitology, Recife, PE 50670-420, Brazil; braga@cpqam.fiocruz.br

**Keywords:** dengue, zika, chikungunya, coronavirus, co-endemic

## Abstract

In this Editorial, we list and discuss some of the main challenges faced by the population and public health authorities in Brazil concerning arbovirus infections, including the occurrence of concurrent epidemics like the ongoing SARS-CoV-2/COVID-19 pandemic.

Optimal ecological and environmental conditions support year-long breeding of mosquito vectors of arboviruses in several Brazilian States. This, combined with socioeconomical factors that facilitate mosquito breeding (e.g., intermittent water supply that leads to short-term water storage in open-air artificial containers) and human exposure to mosquito bites, fosters cyclic and intense transmission of arboviruses in Brazil. In urban and peri-urban areas, the four dengue virus serotypes (DENV1-4), Zika virus (ZIKV), and chikungunya virus (CHIKV) are the most widespread and impactful mosquito-borne pathogens, all transmitted by the highly urbanized and anthropophagic *Aedes aegypti*. Arboviral diseases impose a great health burden to the population in Brazil and represent a constant challenge to health authorities. Diagnosis (and therefore clinical management) and notification of arboviral infections in co-endemic places (where more than one arbovirus co-circulate) are extremely complex. Point-of-care virus-specific testing is non-existent in the public and private health care sectors, and the most important diagnosis is clinical-epidemiological, upon which a case is notified to health authorities as suspected or confirmed based on the Ministry of Health case definitions (which uses clinical symptoms and blood test results, such as platelet counts). However, diseases caused by DENV, ZIKV and CHIKV can lead to similar acute symptoms that may differ only in time of onset, duration and severity [1]—thus, only well-trained, experienced physicians are more apt to correctly diagnose a patient, but even these professionals can misdiagnose without available virus-specific tests. Arboviral diseases are nationally notifiable diseases in Brazil, but in the public sector, which exclusively serves more than 70% of the Brazilian population, only a small proportion of cases notified by health care units undergo confirmatory tests in public reference laboratories through virus-specific molecular or serological assays, or virus culture. For instance, among the notified dengue cases in 2020 (until April), only approximately 23% were tested in reference laboratories [2]. In addition, for DENV and ZIKV, cross-reactivity of serological assays represents a serious issue as it can lead to erroneous results [3]. Official government notifications may thus be biased by inaccurate clinical diagnosis and cross-reactive serological results, and clinical management of infections may not be appropriate if the wrong diagnosis is made. The different socioeconomic realities of Brazilian States also contribute to inconsistencies of arboviral disease notifications.

The cross-reactivity between DENV and ZIKV serological assays are due to similar antigenic regions of viral proteins of these genetically related flaviviruses that can be recognized by the same antibodies. Besides being an issue in serological tests, cross-reactive DENV and ZIKV immunity can have important epidemiological implications in places where these viruses co-circulate. For instance, in vitro, in vivo and epidemiological studies have shown that pre-existing DENV immunity can either protect or enhance ZIKV infection, and consequently impact disease development [4,5,6]. Other studies suggest that the atypically low dengue incidence observed after the Zika epidemics in Brazil and other Latin American countries was due, in part, to short-term DENV protection from ZIKV infections [7,8]. Importantly, this lower dengue incidence was followed by a significant increase in dengue cases [2,7]. The impact of pre-existing DENV and ZIKV immunity in further heterologous infections and, importantly, in clinical diseases, needs to be continuously assessed in endemic areas.

It is also possible that ZIKV or other arboviruses may establish sylvatic transmission cycles in Brazil, as discussed by other authors [9]. If one looks at the map of Paulista, for example, a municipality within the Recife Metropolitan Region (RMR) in Pernambuco State that was heavily affected by ZIKV and CHIKV, forested areas surround all the urban areas where the viruses co-circulated and human cases were concentrated in 2015-16 (Figure 1 and [10]). These forested areas may harbor several sylvatic mosquitoes like *Aedes albopictus*, *Haemagogus janthinomys*, and *Sabethes tarsopus* that feed on non-human primates (NHPs) and may serve as vectors of arboviruses [11]. In addition, NHPs like the common marmoset *Callithrix jacchus* are abundant in the area [12] and found near humans. Importantly, ZIKV RNA and antibodies against several arboviruses have been found in NHPs in different regions of Brazil, including marmosets [13,14,15,16]. The seriousness of an established sylvatic arbovirus transmission cycle in NHPs and sylvatic mosquitoes in Brazil is well represented by yellow fever virus (YFV), which causes sporadic spillover human outbreaks leading to hundreds of deaths. Although a few studies have found little evidence of sylvatic ZIKV transmission in Brazil [16,17], the possibility of a sylvatic cycle being established in distinct Brazilian regions and at different times cannot be excluded. Further governmental or research-related arbovirus surveillance activities should intensify monitoring of sylvatic mosquitoes, NHPs and other small mammals, as the establishment of sylvatic cycles will require changes in the design of control programs.

The ZIKV outbreaks that occurred in Brazil in 2014-16 probably ceased due to herd immunity—however, instead of disappearing, the virus is still circulating in areas that were intensely affected, like the RMR, even if at low rates. In addition, virus transmission during the outbreaks was focal across metropolitan regions, where some areas were more intensely hit than others within the same municipality [4], corroborating the notion of clustered household/community transmission of arboviruses transmitted by *Ae. aegypti*. The low but constant circulation of ZIKV, the presence of prior virus foci with surrounding patchy areas containing higher numbers of naïve people, and the possibility of a sylvatic cycle being established in some regions increase the chances of unexpected re-emergence of the virus. It will also be important to assess the importance of sexual transmission among the sustained, low ZIKV circulation in endemic regions, as the epidemiological relevance of ZIKV sexual transmission may be higher than previously thought ([18] and Magalhaes et al., unpublished).

Escalating the problem of arboviral disease surveillance and management, concurrent outbreaks/epidemics of arboviruses and non-arthropod-borne pathogens can further complicate clinical diagnosis and completely overwhelm/saturate the health care system, as we may be seeing now with the pandemic of coronavirus disease (COVID-19) caused by severe acute respiratory syndrome coronavirus 2 (SARS-CoV-2). The number of notified dengue, Zika and chikungunya cases in Brazil in 2020 have reached over 660,000 by April [2], reflecting a difficult year for arboviral diseases in the country (Figure 2A). Although the true incidences of SARS-CoV-2 infections and COVID-19 cases are unknown in Brazil due to the very limited testing (currently, the Brazilian government recommends that only severe cases are tested in health clinics and hospitals), the notified numbers of infections and deaths are starting to increase, indicating a worsening epidemic scenario as of April 2020 (Figure 2B) [2]. At the moment, health care units like the local rapid-access units (Unidades de Pronto Atendimento-UPAs), which serve communities like the Paulista population (~330,000 habitants), are working with a reduced number of staff as some individuals have fallen ill and many elderly professionals or those with comorbidities are on leave due to fear of becoming infected with the virus. In a recent serosurvey of SARS-CoV-2 antibodies among health professionals in Pernambuco State, 60% have tested positive, confirming these professionals are under very high risk of infection [19]. Although the highest numbers of notified arboviral diseases seemed to have occurred in March 2020, it is very likely that case notification has dropped as a result of fewer people infected with arboviruses seeking health facilities due to the SARS-CoV-2 pandemic. In fact, it would be important to see if household mosquito transmission of arboviruses increases because of social isolation during the COVID-19 pandemic, considering the endophilic behavior of *Ae. aegypti* (although social isolation is necessary, it is also important to assess its effects on other health factors). The blunt reality is that health care units have been dealing with a peak in arbovirus infections and COVID-19 cases concomitantly. Besides the many troubles inherent to an overwhelmed health care system, concurrent epidemics also can complicate clinical-epidemiological diagnoses. Some studies show that dengue cases can be misdiagnosed as respiratory infections and vice-versa [20,21]. Coinfections during concurrent epidemics must also be considered as they may worsen clinical diseases. Coinfections of influenza virus and DENV have been identified in several occasions during concurrent epidemics [22,23,24]. Future control efforts and programs must consider concurrent epidemics as they will most likely continue to happen in the future (e.g., epidemics of DENV and new strains of influenza virus).

## Conclusions

Effective management of arboviral diseases in Brazil requires confronting major challenges. The co-endemicity of multiple and related arboviruses complicates clinical-epidemiological diagnoses, clinical management and case notification, in addition to impacting the epidemiology of arboviral diseases in unclear ways. The possible establishment of sylvatic transmission cycles will represent a significant additional challenge to the development of control programs and should be constantly monitored. Lastly, concurrent epidemics like the SARS-CoV-2/COVID-19 or other respiratory pathogens/illnesses can overwhelm health care systems and further complicate clinical-epidemiological diagnoses. Efforts to better control these diseases must seriously consider all these issues.

## Figures and Tables

**Figure 1:**
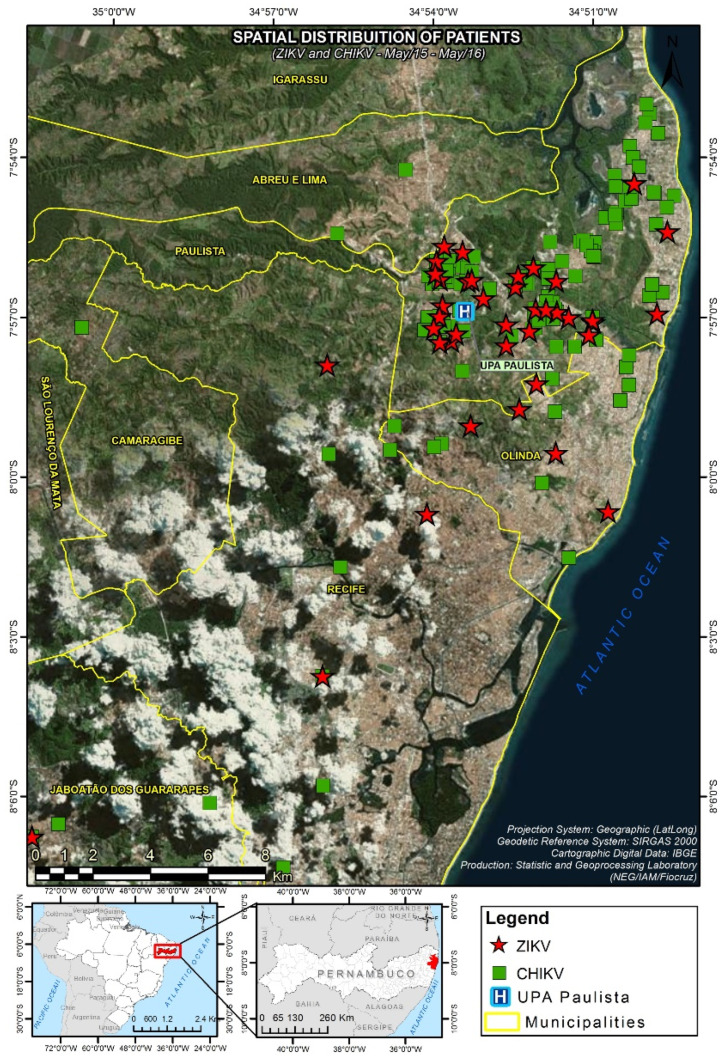
Mapped cases of Zika virus (ZIKV) and chikungunya virus (CHIKV) infections in Paulista, Pernambuco State, Brazil, in 2015-16. Note the green/forested areas surrounding the urban areas where cases were concentrated: an optimal interface for the establishment of sylvatic cycles of arbovirus transmission (this map was published in [10]).

**Figure 2:**
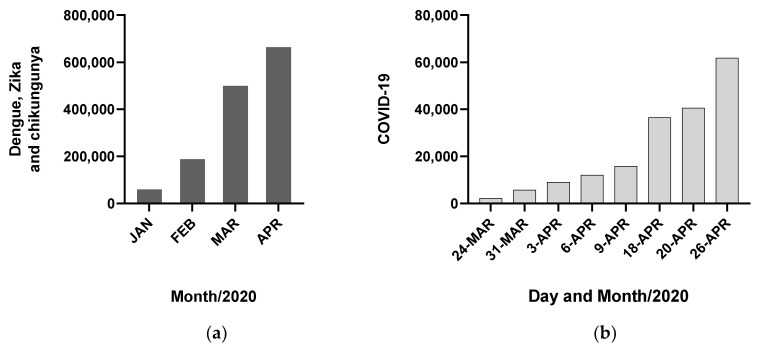
Cumulative notified cases of dengue, Zika and chikungunya (**a**) and COVID-19 (**b**) in Brazil as of April 2020.

## References

[1] Brito C.A., Cordeiro M.T. (2016). One year after the Zika virus outbreak in Brazil: From hypotheses to evidence. Rev. Soc. Bras. Med. Trop..

[2] Ministério da Saúde do Brasil Boletins Epidemiológicos. https://www.saude.gov.br/boletins-epidemiologicos.

[3] Zaidi M.B., Cedillo-Barron L., Gonzalez Y.A.M.E., Garcia-Cordero J., Campos F.D., Namorado-Tonix K., Perez F. (2020). Serological tests reveal significant cross-reactive human antibody responses to Zika and Dengue viruses in the Mexican population. Acta Trop..

[4] Rodriguez-Barraquer I., Costa F., Nascimento E.J.M., Nery N.J., Castanha P.M.S., Sacramento G.A., Cruz J., Carvalho M., De Olivera D., Hagan J.E. (2019). Impact of preexisting dengue immunity on Zika virus emergence in a dengue endemic region. Science.

[5] Oliveira R.A., de Oliveira-Filho E.F., Fernandes A.I., Brito C.A., Marques E.T., Tenorio M.C., Gil L.H. (2019). Previous dengue or Zika virus exposure can drive to infection enhancement or neutralisation of other flaviviruses. Mem. Inst. Oswaldo Cruz.

[6] Watanabe S., Tan N.W.W., Chan K.W.K., Vasudevan S.G. (2019). Dengue Virus and Zika Virus Serological Cross-reactivity and Their Impact on Pathogenesis in Mice. J. Infect. Dis..

[7] Borchering R.K., Huang A.T., Mier Y.T.-R.L., Rojas D.P., Rodriguez-Barraquer I., Katzelnick L.C., Martinez S.D., King G.D., Cinkovich S.C., Lessler J. (2019). Impacts of Zika emergence in Latin America on endemic dengue transmission. Nat. Commun..

[8] Perez F., Llau A., Gutierrez G., Bezerra H., Coelho G., Ault S., Barbiratto S.B., de Resende M.C., Cerezo L., Kleber G.L. (2019). The decline of dengue in the Americas in 2017: Discussion of multiple hypotheses. Trop. Med. Int. Health.

[9] Figueiredo L.T.M. (2019). Human Urban Arboviruses Can Infect Wild Animals and Jump to Sylvatic Maintenance Cycles in South America. Front. Cell. Infect. Microbiol..

[10] Magalhaes T., Braga C., Cordeiro M.T., Oliveira A.L.S., Castanha P.M.S., Maciel A.P.R., Amancio N.M.L., Gouveia P.N., Peixoto-da-Silva V.J., Peixoto T.F.L. (2017). Zika virus displacement by a chikungunya outbreak in Recife, Brazil. PLoS Negl. Trop. Dis..

[11] Aragao N.C., Muller G.A., Balbino V.Q., Costa Junior C.R., Figueiredo Junior C.S., Alencar J., Marcondes C.B. (2010). A list of mosquito species of the Brazilian State of Pernambuco, including the first report of Haemagogus janthinomys (Diptera: Culicidae), yellow fever vector and 14 other species (Diptera: Culicidae). Rev. Soc. Bras. Med. Trop..

[12] Thompson C.L., Robl N.J., Melo L.C.O., Valença-Montenegro M.M., Valle Y.B.M., Oliveira M.A.B., Vinyard C.J. (2013). Spatial distribution and exploitation of trees gouged by common marmosets (*Callithrix jacchus*). Int. J. Primatol..

[13] Terzian A.C.B., Zini N., Sacchetto L., Rocha R.F., Parra M.C.P., Del Sarto J.L., Dias A.C.F., Coutinho F., Rayra J., da Silva R.A. (2018). Evidence of natural Zika virus infection in neotropical non-human primates in Brazil. Sci. Rep..

[14] Favoretto S.R., Araujo D.B., Duarte N.F.H., Oliveira D.B.L., da Crus N.G., Mesquita F., Leal F., Machado R.R.G., Gaio F., Oliveira W.F. (2019). Zika Virus in Peridomestic Neotropical Primates, Northeast Brazil. Ecohealth.

[15] De Oliveira-Filho E.F., Oliveira R.A.S., Ferreira D.R.A., Laroque P.O., Pena L.J., Valenca-Montenegro M.M., Mota R.A., Gil L. (2018). Seroprevalence of selected flaviviruses in free-living and captive capuchin monkeys in the state of Pernambuco, Brazil. Transbound. Emerg. Dis..

[16] Moreira-Soto A., Carneiro I.O., Fischer C., Feldmann M., Kummerer B.M., Silva N.S., Santos U.G., Souza B., Liborio F.A., Valenca-Montenegro M.M. (2018). Limited Evidence for Infection of Urban and Peri-urban Nonhuman Primates with Zika and Chikungunya Viruses in Brazil. mSphere.

[17] Pauvolid-Correa A., Goncalves Dias H., Marina Siqueira Maia L., Porfirio G., Oliveira Morgado T., Sabino-Santos G., Helena Santa Rita P., Teixeira Gomes Barreto W., Carvalho de Macedo G., Marinho Torres J. (2019). Zika Virus Surveillance at the Human-Animal Interface in West-Central Brazil, 2017–2018. Viruses.

[18] Rosenberg E.S., Doyle K., Munoz-Jordan J.L., Klein L., Adams L., Lozier M., Weiss K., Sharp T.M., Paz-Bailey G. (2019). Prevalence and Incidence of Zika Virus Infection Among Household Contacts of Patients With Zika Virus Disease, Puerto Rico, 2016–2017. J. Infect. Dis..

[19] Centro de Informações Estratégicas de Vigilância em Saúde de Pernambuco (CIEVS). https://www.cievspe.com/novo-coronavirus-2019-ncov.

[20] Chacon R., Clara A.W., Jara J., Armero J., Lozano C., El Omeiri N., Widdowson M.A., Azziz-Baumgartner E. (2015). Influenza Illness among Case-Patients Hospitalized for Suspected Dengue, El Salvador, 2012. PLoS ONE.

[21] Restrepo B.N., Piedrahita L.D., Agudelo I.Y., Parra-Henao G., Osorio J.E. (2012). Frequency and clinical features of dengue infection in a schoolchildren cohort from medellin, Colombia. J. Trop. Med..

[22] Perez M.A., Gordon A., Sanchez F., Narvaez F., Gutierrez G., Ortega O., Nunez A., Harris E., Balmaseda A. (2010). Severe coinfections of dengue and pandemic influenza A H1N1 viruses. Pediatr. Infect. Dis. J..

[23] Lopez Rodriguez E., Tomashek K.M., Gregory C.J., Munoz J., Hunsperger E., Lorenzi O.D., Irizarry J.G., Garcia-Gubern C. (2010). Co-infection with dengue virus and pandemic (H1N1) 2009 virus. Emerg. Infect. Dis..

[24] Hussain R., Al-Omar I., Memish Z.A. (2012). The diagnostic challenge of pandemic H1N1 2009 virus in a dengue-endemic region: A case report of combined infection in Jeddah, Kingdom of Saudi Arabia. J. Infect. Public Health.

